# Influence of the COVID-19 Pandemic on Overall Physician Visits and Telemedicine Use Among Patients With Type 1 or Type 2 Diabetes in Japan

**DOI:** 10.2188/jea.JE20220032

**Published:** 2022-10-05

**Authors:** Susumu Yagome, Takehiro Sugiyama, Kosuke Inoue, Ataru Igarashi, Ryotaro Bouchi, Mitsuru Ohsugi, Kohjiro Ueki, Atsushi Goto

**Affiliations:** 1 Department of Health Data Science, Yokohama City University Graduate School of Data Science, Yokohama, Japan; 2 Integrity Healthcare Co., Ltd., Tokyo, Japan; 3 Diabetes and Metabolism Information Centre, National Centre for Global Health and Medicine, Tokyo, Japan; 4 Department of Health Services Research, Faculty of Medicine, University of Tsukuba, Ibaraki, Japan; 5 Department of Epidemiology, UCLA Fielding School of Public Health, Los Angeles, CA, USA; 6 Department of Social Epidemiology, Graduate School of Medicine, Kyoto University, Kyoto, Japan; 7 Unit of Public Health and Preventive Medicine, Yokohama City University School of Medicine, Kanagawa, Japan; 8 Department of Health Economics and Outcomes Research, Graduate School of Pharmaceutical Sciences, the University of Tokyo, Tokyo, Japan; 9 Department of Diabetes, Endocrinology and Metabolism, National Centre for Global Health and Medicine, Tokyo, Japan; 10 Diabetes Research Centre, National Centre for Global Health and Medicine, Tokyo, Japan

**Keywords:** COVID-19, telemedicine, outpatient, diabetes

## Abstract

**Background:**

Regular visits with healthcare professionals are important for preventing serious complications in patients with diabetes. The purpose of this retrospective cohort study was to clarify whether there was any suppression of physician visits among patients with diabetes during the spread of the novel coronavirus 2019 (COVID-19) in Japan and to assess whether telemedicine contributed to continued visits.

**Methods:**

We used the JMDC Claims database, which contains the monthly claims reported from July 2018 to May 2020 and included 4,595 (type 1) and 123,686 (type 2) patients with diabetes. Using a difference-in-differences analysis, we estimated the changes in the monthly numbers of physician visits or telemedicine per 100 patients in April and May 2020 compared with the same months in 2019.

**Results:**

For patients with type 1 diabetes, the estimates for total overall physician visits were −2.53 (95% confidence interval [CI], −4.63 to 0.44) in April and −8.80 (95% CI, −10.85 to −6.74) in May; those for telemedicine visits were 0.71 (95% CI, 0.47–0.96) in April and 0.54 (95% CI, 0.32–0.76) in May. For patients with type 2 diabetes, the estimates for overall physician visits were −2.50 (95% CI, −2.95 to −2.04) in April and −3.74 (95% CI, −4.16 to −3.32) in May; those for telemedicine visits were 1.13 (95% CI, 1.07–1.20) in April and 0.73 (95% CI, 0.68–0.78) in May.

**Conclusion:**

The COVID-19 pandemic was associated with suppression of physician visits and a slight increase in the utilization of telemedicine among patients with diabetes during April and May 2020.

## INTRODUCTION

Diabetes mellitus is a prevalent, chronic disease; a substantial increase in the prevalence of the disease is expected over the next decades in Japan.^1^^,^^2^ Regular patient visits with their healthcare providers are deemed to be necessary in order to prevent serious complications among patients with diabetes.^3^^,^^4^ However, in response to the spread of the novel coronavirus 2019 (COVID-19), lockdowns and requests/orders to remain at home were implemented in many jurisdictions globally. This raised concerns that patients with chronic diseases, such as diabetes, might not be able to effectively continue their treatment due to the restricted physical interactions with physicians during the pandemic.^5^

To prevent the spread of the responsible virus, the Japanese government declared the first COVID-19-related state of emergency for seven prefectures (Tokyo, Kanagawa, Saitama, Chiba, Osaka, Hyogo, and Fukuoka) on April 7, 2020, expanding to cover the entire country on April 16, 2020; the state of emergency was lifted on May 25, 2020. At the same time, telemedicine regulations were significantly relaxed to ensure the continuity of some type of physician–patient interaction.^6^ Previously, the conditions for telemedicine access were limited to patients with certain diseases, such as headaches and incurable diseases. However, beginning on April 10, 2020, all patients were permitted to receive care via telemedicine if the doctor deemed it necessary.

Previous studies reported decreased face-to-face care and increased utilization of telemedicine during the spread of COVID-19 in the United States.^7^^–^^10^ In addition, diabetes mellitus is known to be a risk factor for increased mortality due to COVID-19,^11^ and patients may have refrained from face-to-face care to prevent infection with COVID-19. Furthermore, declining trends in diabetes-related medical care during the period of infection spread in Japan were reported.^12^ Additionally, changes in lifestyles and their associations with metabolic and glycemic status among patients with diabetes were reported after the first COVID-19-related state of emergency in Japan.^13^ However, to the best of our knowledge, no studies have investigated the impact of COVID-19 pandemic on changes in physician visits or telemedicine visits in April and May 2020 as compared to the same months in 2019 among patients with diabetes. Clarifying whether, and to what extent, the COVID-19 pandemic influenced diabetes care through the possible suppression of physician visits and greater reliance on telemedicine is essential. In addition, because type 1 and type 2 diabetes have different patient demographics, the impact of the COVID-19 pandemic on visits might differ between these patient groups. Thus, each type of patient population needs to be investigated separately.

The purpose of this study was to assess changes in overall physician visits and telemedicine use for diabetes care during the COVID-19 pandemic in Japan.

## METHODS

### Study population

We obtained anonymized individual data from the JMDC Claims Database, a medical database managed by JMDC Inc. Japan has adopted a universal health insurance system, and citizens are enrolled in several insurance systems, including employees’ health insurance programs, the national health insurance program, and the elderly health care system. The JMDC database contains the monthly claims reported by multiple employee health insurance programs since January 2005, covering 10,502,630 beneficiaries (either the insured persons or their dependents) as of May 2020. It includes information, such as age, sex, details of medical procedures performed, International Classification of Diseases (ICD-10) diagnosis codes, medical care received, consultations, and medications prescribed. This study was approved by the institutional review board of the Yokohama City University (Reference No. B200800024). Because data were anonymized, the requirement for informed consent was waived for this study.

A total of 128,281 people who met the criteria of having diabetes during the baseline period (July 2018 to December 2018) and had continued observation periods between July 2018 and May 2020 were included in the study.

### Covariates

The presence of hypertension and dyslipidemia was defined based on the medications that were prescribed (eTable 1). Illness severity was assessed using the Charlson Comorbidities Index,^14^ Elixhauser Index,^14^ and Diabetes Complications Severity Index.^15^ The Charlson Comorbidities Index and Elixhauser Index require three-digit ICD-10 codes; however, some ICD-10 codes in the JMDC database had only two digits. Therefore, there were patients with diabetes who only had two-digit diabetes-related ICD-10 codes and did not have ICD-10 codes that were used to count diabetes in the Charlson Comorbidities Index and the Elixhauser Index.

### Diabetes definitions

Type 1 or type 2 diabetes was defined based on the validated definitions reported in previous studies.^16^^–^^18^ Patients were classified as having type 1 diabetes if their record had an ICD-10 code starting with E10 or O240 (patients with ‘unstable diabetes’ were excluded), they were prescribed insulin (World Health Organization Anatomical Therapeutic Chemical [WHO-ATC] Classification code starting with A10A) during the baseline period, and their record had a medical practice code for self-monitoring of blood glucose levels (eTable 2).^16^ Patients were defined as having type 2 diabetes if their record included an ICD-10 code starting with E11 or E14, had at least one prescription for an antidiabetic medication (WHO-ATC Classification code starting with A10, excluding those starting with A10X), and did not satisfy the above-mentioned criteria for type 1 diabetes during the baseline period.^17^^,^^18^

### Outcomes

The outcomes of the current study were monthly number of physician visits (including both face-to-face visits and telemedicine visits) with prescriptions of antidiabetic medication(s) and monthly number of telemedicine visits with prescriptions of antidiabetic medication(s) (eTable 3).

### Statistical analyses

A difference-in-difference analysis was used to compare the difference between the average number of overall physician visits or telemedicine visits during January, February, and March and the number of physician visits or telemedicine visits in April or May in 2020 with the corresponding difference in 2019. To examine the effect of the COVID-19 pandemic on physician visits, we need to compare the number of visits that would have been observed in the absence of COVID-19 in 2020 (unobserved counterfactual) with the number observed in 2020 during the COVID-19 infection epidemic. Because it is not possible to measure unobserved counterfactual, we utilized the difference-in-difference design to substitute the unobserved number with the trends observed in the year 2019, assuming that in the absence of COVID-19 in 2020, the difference in the number of visits between the year 2019 and the year 2020 is constant over time (a parallel time trend assumption). The dependent variable was the monthly number of overall physician visits or telemedicine visits with diabetes prescriptions per 100 people with diabetes (eTable 3), and the independent variables included indicator variables representing i) January–March, ii) April, iii) May, and iv) year 2020, and product terms for April or May and the year indicator. The product terms represent the effect estimates of the COVID-19 pandemic on physician visits in April or May 2020: ([number of visits in April or May 2020] − [average number of visits per month between January and March 2020]) − ([number of visits in April or May 2019] − [average number of visits per month between January and March 2019]). For subgroup analyses, we categorized participants according to age categories (0–19 years [for type 1 diabetes only], 20–39 years, 40–59 years, ≥60 years [for type 1 diabetes only], 60–69 years [for type 2 diabetes only], and ≥70 years [for type 2 diabetes only]) and sex; difference-in-difference analyses were conducted for these subgroups. A stratified analysis was performed for each complication as defined by the Diabetes Complication Severity Index score.

To assess whether trends during the pre-intervention periods (January–March) differ between 2019 and 2020, we calculated β coefficients and 95% confidence intervals (CIs) for interaction by including an ordinal variable representing January–March, an indicator variable for the year 2020, and their product term (ie, interaction term) into liner regression models, with cluster–robust standard errors with individuals treated as clusters using the lm_robust function from the estimatr package in R (R Foundation for Statistical Computing, Vienna, Austria).^19^^,^^20^ However, linear regression models might yield biased results, as count variables cannot have values less than zero. Therefore, as sensitivity analyses, we also fitted negative binomial regression models, with identity link function and cluster-robust standard errors with individuals treated as clusters, using the hurdle function from the pscl package and the VCovCL function from the sandwich package in R.^21^^,^^22^

All statistical analyses were performed using R software (version 4.05 for Windows), with a two-sided *P*-value <0.05 being regarded as statistically significant.

## RESULTS

The study included 4,595 patients with type 1 diabetes and 123,686 patients with type 2 diabetes. The participant characteristics are presented in Table 1. Among 4,595 patients with type 1 diabetes, the median age was 45 (interquartile range [IQR], 34–53) years, 2,483 (54.0%) were men, 918 (20.0%) had hypertension, 918 (21.2%) had dyslipidemia, the prevalence of microvascular complications ranged from 13.7–36.9%, and 3.7–10.2% had macrovascular complications. Among 123,686 patients with type 2 diabetes, the median age was 55 (IQR, 49–61) years, 92,630 (74.9%) were men, 68,421 (55.3%) had hypertension, 66,604 (53.8%) had dyslipidemia, 6.7–16.7% had microvascular complications, and 2.6–19.2% had macrovascular complications.

**Table 1.** Participant characteristics, according to diabetes type

Characteristic	Type 1 diabetes (*n* = 4,595)	Type 2 diabetes (*n* = 123,686)
Age, years, median (IQR)	45 (34–53)	55 (49–61)
Men, *n* (%)	2,483 (54.0%)	92,630 (74.9%)
Hypertension, *n* (%)	918 (20.0%)	68,421 (55.3%)
Hyperlipidemia, *n* (%)	975 (21.2%)	66,604 (53.8%)
Charlson Comorbidities Index, median (IQR)	2 (1–3)	1 (0–3)
Elixhauser Index, median (IQR)	2 (1–3)	2 (1–3)
Diabetes Complications Severity Index, median (IQR)	1 (0–2)	0 (0–2)
Microvascular complications		
Retinopathy,^a^ *n* (%)	1,695 (36.9%)	24,237 (15.6%)
Nephropathy,^a^ *n* (%)	929 (20.2%)	20,698 (16.7%)
Neuropathy,^a^ *n* (%)	629 (13.7%)	8,229 (6.7%)
Macrovascular complications		
Cerebrovascular,^a^ *n* (%)	233 (5.0%)	10,421 (8.4%)
Cardiovascular,^a^ *n* (%)	469 (10.2%)	23,771 (19.2%)
Peripheral vascular disease,^a^ *n* (%)	170 (3.7%)	3,267 (2.6%)
Metabolic complications,^a^ *n* (%)	600 (13.1%)	667 (0.5%)

^a^Components of Diabetes Complications Severity Index.

**Figure 1.**
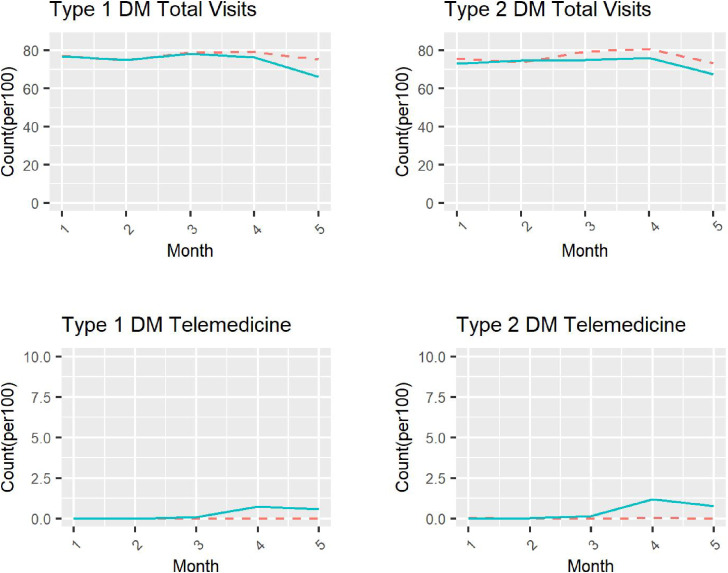
Changes in overall visits and telemedicine use among patients with diabetes from January to May, 2019 and 2020 Solid line shows the number of visits/100 population in 2020. Dashed line shows the number of visits/100 population in 2019.

Regarding the trends during the pre-intervention periods between year 2019 and year 2020, for patients with type 1 diabetes, the coefficient of the interaction term was −0.13 (95% CI, −1.30 to 1.04) for the total overall physician visits and 0.04 (95% CI, 0.00–0.09) for telemedicine use; for patients with type 2 diabetes, the coefficient of the interaction term was −0.98 (95% CI, −1.24 to −0.73) for the total overall physician visits and 0.07 (95% CI, 0.06–0.08) for telemedicine use (Table 2 and Table 3). The medical procedure counts for each month are shown in Figure 1. Counts were performed on a per 100 people with diabetes basis to allow for comparisons (eTable 4 and eTable 5). For patients with type 1 diabetes (Table 2), the difference-in-difference estimates for total overall physician visits were −2.53 (95% CI, −4.63 to 0.44) in April and −8.80 (95% CI, −10.85 to −6.74) in May; those for telemedicine visits were 0.71 (95% CI, 0.47–0.96) in April and 0.54 (95% CI, 0.32–0.76) in May. For patients with type 2 diabetes (Table 3), the difference-in-difference estimates for total overall physician visits were −2.50 (95% CI, −2.95 to −2.04) in April and −3.74 (95% CI, −4.16 to −3.32) in May; those for telemedicine visits were 1.13 (95% CI, 1.07–1.20) in April and 0.73 (95% CI, 0.68–0.78) in May. Among patients with either type 1 and type 2 diabetes, women tended to refrain from physician visits more often than men (Table 2 and Table 3). The results of the stratified analysis by Diabetes Complication Severity Index score are shown in the eTable 6 and eTable 7. In strata with diabetes complications, trends toward a decrease in the total number of physician visits and a slight increase in the number of telemedicine visits were observed. The negative binomial regression models with cluster-robust standard errors yielded results nearly identical to the linear regression models (eTable 8 and eTable 9). For telemedicine use, the negative binomial regression with identity link function did not converge, possibly because of few counts per month. Therefore, results from the negative binomial regression were only shown for overall visits.

**Table 2.** Difference-in-difference model: Total and telemedicine visits 2020 vs 2019 for patients with type 1 diabetes

	Number of visits per 100 people with diabetes (95% confidence interval)^a^								

	Overall (*n* = 4,582)			Men (*n* = 2,473)			Women (*n* = 2,109)		

	Preintervention periods^b^	Apr^a^	May^a^	Preintervention periods^b^	Apr^a^	May^a^	Preintervention periods^b^	Apr^a^	May^a^
**Total overall physician visits**									
Total	−0.13 (−1.30 to 1.04)	−2.53 (−4.63 to −0.44)	−8.80 (−10.85 to −6.74)	0.18 (−1.42 to 1.79)	−3.53 (−6.44 to −0.62)	−5.51 (−8.34 to −2.68)	−0.50 (−2.21 to 1.22)	−1.36 (−4.38 to 1.66)	−12.64 (−15.61 to −9.68)
1–19 years	−1.94 (−5.16 to 1.28)	−7.95 (−13.45 to −2.44)	−15.50 (−21.65 to −9.36)	−0.24 (−5.40 to 4.92)	−8.61 (−17.00 to −0.23)	−11.00 (−20.59 to −1.42)	−3.09 (−7.23 to 1.04)	−7.49 (−14.82 to −0.17)	−18.57 (−26.60 to −10.53)
20–39 years	−0.32 (−2.84 to 2.21)	−0.69 (−4.81 to 3.43)	−8.47 (−12.65 to −4.29)	0.00 (−3.44 to 3.44)	−2.06 (−7.84 to 3.71)	−5.50 (−11.35 to 0.35)	−0.67 (−4.39 to 3.05)	0.83 (−5.07 to 6.72)	−11.77 (−17.74 to −5.80)
40–59 years	−0.36 (−1.98 to 1.25)	−2.73 (−5.68 to 0.21)	−6.94 (−9.72 to −4.15)	−0.03 (−2.18 to 2.11)	−3.71 (−7.65 to 0.24)	−4.48 (−8.19 to −0.76)	−0.81 (−3.27 to 1.64)	−1.40 (−5.82 to 3.01)	−10.30 (−14.51 to −6.10)
≧60 years	3.40 (0.25–6.55)	0.07 (−6.63 to 6.77)	−11.89 (−18.26 to −5.52)	2.18 (−2.26 to 6.63)	−1.72 (−11.26 to 7.82)	−6.88 (−16.15 to 2.39)	4.72 (0.23–9.21)	2.00 (−7.47 to 11.47)	−17.31 (−26.02 to −8.60)

**Telemedicine**									
Total	0.04 (0.00–0.09)	0.71 (0.47–0.96)	0.54 (0.32–0.76)	0.02 (−0.02 to 0.06)	0.59 (0.29–0.90)	0.63 (0.32–0.95)	0.07 (−0.01 to 0.15)	0.85 (0.45–1.25)	0.43 (0.13–0.73)
1–19 years	0.10 (−0.09 to 0.29)	0.71 (−0.06 to 1.48)	0.52 (−0.15 to 1.19)	0.00 (0.00–0.00)	1.44 (−0.19 to 3.06)	0.96 (−0.38 to 2.29)	0.16 (−0.16 to 0.48)	0.22 (−0.46 to 0.89)	0.22 (−0.46 to 0.89)
20–39 years	0.05 (−0.04 to 0.13)	0.69 (0.19–1.20)	0.69 (0.19–1.20)	0.09 (−0.08 to 0.25)	0.46 (−0.14 to 1.05)	0.97 (0.14–1.81)	0.00 (0.00–0.00)	0.95 (0.12–1.79)	0.38 (−0.15 to 0.91)
40–59 years	0.04 (−0.02 to 0.10)	0.78 (0.43–1.13)	0.54 (0.24–0.84)	0.00 (0.00–0.00)	0.56 (0.17–0.95)	0.49 (0.13–0.85)	0.10 (−0.04 to 0.23)	1.08 (0.45–1.72)	0.61 (0.10–1.11)
≧60 years	0.00 (0.00–0.00)	0.41 (−0.16 to 0.99)	0.21 (−0.20 to 0.61)	0.00 (0.00–0.00)	0.40 (−0.39 to 1.18)	0.40 (−0.39 to 1.18)	0.00 (0.00–0.00)	0.43 (−0.42 to 1.28)	0.00 (0.00–0.00)

^a^The estimates for April or May 2020 represent ([number of visits in April or May 2020] − [average number of visits er month between January and March 2020]) − ([number of visits in April or May 2019] − [average number of visits er month between January and March 2019]).

^b^The preintervention difference in linear trend between year 2019 and year 2020.

**Table 3.** Difference-in-difference model: Total and telemedicine visits 2020 vs 2019 for patients with type 2 diabetes

	Number of visits per 100 people with diabetes (95% confidence interval)^a^								

	Overall (*n* = 120,631)			Men (*n* = 90,515)			Women (*n* = 30,116)		

	Preintervention periods^b^	Apr^a^	May^a^	Preintervention periods^b^	Apr^a^	May^a^	Preintervention periods^b^	Apr^a^	May^a^
**Total overall physician visits**									
Total	−0.98 (−1.24 to −0.73)	−2.50 (−2.95 to −2.04)	−3.74 (−4.16 to −3.32)	−1.15 (−1.44 to −0.86)	−2.23 (−2.75 to −1.70)	−2.54 (−3.02 to −2.05)	−0.49 (−0.99 to 0.01)	−3.32 (−4.22 to −2.42)	−7.36 (−8.20 to −6.53)
20–39 years	0.60 (−0.55 to 1.75)	−2.21 (−4.24 to −0.18)	1.02 (−0.87 to 2.92)	0.47 (−0.85 to 1.79)	−2.20 (−4.54 to 0.13)	1.80 (−0.38 to 3.99)	0.98 (−1.37 to 3.33)	−2.23 (−6.35 to 1.88)	−1.22 (−4.99 to 2.55)
40–59 year	−1.32 (−1.64 to −1.00)	−2.04 (−2.61 to −1.48)	−2.77 (−3.30 to −2.25)	−1.50 (−1.86 to −1.14)	−1.74 (−2.38 to −1.10)	−1.42 (−2.02 to −0.83)	−0.71 (−1.36 to −0.06)	−3.06 (−4.23 to −1.89)	−7.31 (−8.40 to −6.22)
60–69 years	−0.63 (−1.11 to −0.15)	−3.35 (−4.24 to −2.46)	−6.37 (−7.17 to −5.57)	−0.78 (−1.35 to −0.21)	−3.25 (−4.31 to −2.20)	−5.70 (−6.65 to −4.75)	−0.25 (−1.15 to 0.66)	−3.60 (−5.24 to −1.96)	−8.07 (−9.57 to −6.57)
≧70 years	0.30 (−0.96 to 1.56)	−4.96 (−7.28 to −2.64)	−9.08 (−11.22 to −6.95)	1.01 (−0.60 to 2.61)	−4.62 (−7.62 to −1.62)	−8.54 (−11.28 to −5.80)	−0.85 (−2.87 to 1.17)	−5.52 (−9.19 to −1.85)	−9.97 (−13.37 to −6.56)

**Telemedicine**									
Total	0.07 (0.06–0.08)	1.13 (1.07–1.20)	0.73 (0.68–0.78)	0.06 (0.05–0.07)	1.09 (1.02–1.16)	0.70 (0.65–0.76)	0.10 (0.08–0.13)	1.27 (1.13–1.40)	0.80 (0.69–0.91)
20–39 years	0.05 (0.00–0.09)	0.80 (0.56–1.03)	0.68 (0.47–0.90)	0.04 (−0.01 to 0.10)	0.59 (0.34–0.84)	0.72 (0.46–0.98)	0.06 (−0.02 to 0.15)	1.39 (0.81–1.97)	0.57 (0.19–0.95)
40–59 years	0.06 (0.05–0.08)	1.06 (0.98–1.13)	0.69 (0.63–0.75)	0.05 (0.04–0.06)	1.01 (0.93–1.09)	0.66 (0.59–0.72)	0.11 (0.07–0.14)	1.22 (1.05–1.39)	0.78 (0.65–0.92)
60–69 years	0.08 (0.06–0.10)	1.33 (1.20–1.46)	0.85 (0.74–0.95)	0.07 (0.04–0.09)	1.32 (1.17–1.47)	0.82 (0.69–0.94)	0.10 (0.05–0.15)	1.33 (1.09–1.58)	0.92 (0.71–1.14)
≧70 years	0.12 (0.04–0.20)	1.66 (1.27–2.04)	0.68 (0.42–0.93)	0.13 (0.02–0.23)	1.90 (1.37–2.42)	0.74 (0.41–1.08)	0.12 (0.00–0.23)	1.27 (0.72–1.82)	0.57 (0.18–0.95)

^a^The estimates for April or May 2020 represent ([number of visits in April or May 2020] − [average number of visits er month between January and March 2020]) − ([number of visits in April or May 2019] − [average number of visits er month between January and March 2019]).

^b^The preintervention difference in linear trend between 2019 and 2020.

## DISCUSSION

In this large-scale, retrospective cohort study of patients with type 1 and type 2 diabetes, the difference-in-difference analyses suggested that the COVID-19 pandemic was associated with suppression of physician visits among patients with either type of disease. Because this study focused on diabetes-specific consultations, rather than all consultations, we were able to examine the impact of the COVID-19 pandemic on continuing treatment, which is important in patients with diabetes. The mean number of overall physician visits, for patients with type 2 diabetes, statistically significantly decreased by 2.50 in April and 3.74 in May. For patients with type 1 diabetes, there was a non-significant decrease in April (2.53) and a statistically significant decrease in May (8.80) for the numbers of overall physician visits. The number of telemedicine visits increased in both April and May for patients with either type 1 or type 2 diabetes; however, both estimates were around 1, suggesting that telemedicine did not greatly contribute to continued physician visits among patients with either type of disease in Japan during the spread of COVID-19 in 2020.

Previous studies have suggested that the spread of COVID-19 suppressed physician visits among general patients (not limited to patients with diabetes).^5^^–^^8^ In addition, a recent retrospective study in Japan reported a slight decrease in physician visits in April 2020 among patients with chronic diseases (*n* = 10,346) including diabetes (*n* = 1,532)^23^; however, the study did not specifically examine the trend among patients with diabetes. In our study, decreases in monthly counts of overall physician visits per 100 patients were greater in patients with type 1 diabetes (8.80) than type 2 diabetes (3.74) in May 2020. A decrease of 8.80 visits in patients with type 1 diabetes might have resulted in the failure to prescribe the necessary insulin, which could have been life-threatening. There is a possibility that telemedicine visits did not greatly increase between 2019 and 2020 because the suppression of physician visits was not considerable. For patients with type 1 diabetes, prescription of blood glucose self-monitoring devices might have been an obstacle to the adoption of telemedicine. Additionally, there are few financial incentives for medical institutions to adopt telemedical visits; therefore, they may not have actively promoted telemedicine. According to the data released by the Ministry of Health, Labour and Welfare, only 50% of Japan’s medical institutions implemented telemedicine visits in April 2020.^24^

The results of the stratified analysis showed that the degree of overall physician visit suppression differed by age and sex. For patients with either type 1 or type 2 diabetes, the number of physician visits decreased more among women and older patients than among men and younger patients, suggesting that women and older patients tend to avoid physician visits, possibly due to a fear of infection. The increase in the number of telemedicine visits observed for people in the older age groups also suggests that the older patients are more likely to choose telemedicine as a means of continuing their physician visits while also reducing the risk of infection. We hypothesized that the working-age population, who are familiar with digital technology, would be the most likely to use telemedicine. However, contrary to our expectations, telemedicine was not widely used by them, compared to other age groups, for both patients with type 1 and type 2 diabetes (Table 2 and Table 3). The results of the stratified analysis by diabetes complication severity index score showed a trend toward a decrease in the total number of physician visits and a slight increase in the number of telemedicine visits in the group with diabetes complications (eTable 6 and eTable 7). These results suggest that women, older patients, and patents with diabetes complications are less likely to seek visits possibly because of their fear of infection.

This study has several limitations. First, the data set used was based on insured persons (including dependents) in Japanese employees’ health insurance programs. Thus, our study population was limited to the working-age population and their dependents because the JMDC database is intended for people with employment insurance, suggesting the need for cautious interpretation of the data. In particular, it should be noted that, in Japan, 50.2% of patients with diabetes are 70 years old or older^25^; however, we were unable investigate the impact of the COVID-19 pandemic among those in this age group. Second, the definitions of diabetes, physician visits, and telemedicine use were based on data in the claims database, which are subject to misclassification. Specifically, patients with type 1 and type 2 diabetes might not have been correctly distinguished in some cases. Third, the first state of emergency declaration due to COVID-19, in Japan, was issued on April 7, 2020 and lifted on May 25, 2020. Thus, only a short period of time was available in which to clarify the impact of COVID-19 on overall physician visits and telemedicine use. Furthermore, although the number of COVID-19 cases were high in major cities, such as Tokyo and Osaka, during April and May 2020, city-specific data were not available in the JMDC database; therefore, we may have underestimated the effect of the COVID-19 pandemic on physician visits and telemedicine use when we focused on the effect especially in the affected areas. Finally, the key assumption of the difference-in-difference design (a parallel time trend assumption) could not be verified. Indeed, there were statistically significant differences, especially for type 2 diabetes possibly because of its large sample size (Table 2 and Table 3). However, because the number of physician visits were largely parallel during the pre-intervention period (January to March) between 2019 and 2020 (Figure 1), although there were small but statistically significant differences (Table 2 and Table 3). Therefore, we believe that the time trends of physician visits in 2020 would have been largely similar to those in 2019, in the absence of COVID-19.

In conclusion, our results suggest that the COVID-19 pandemic was associated with suppression of physician visits and a slight increase in the utilization of telemedicine among patients with diabetes during April and May 2020 in Japan. These results may provide insight into the management of diabetes and to policy evaluations regarding the role of telemedicine.

## SUPPLEMENTARY MATERIAL

eTable 1. Medications prescribed codes (WHO-ATC^a^ Classification code) for hypertension and dyslipidaemia

eTable 2. Medical practice codes for self-monitoring of blood glucose levels in patients with Type 1 diabetes or childhood hypoglycaemia^a^

eTable 3. Medical practice codes for medical visits

eTable 4. Observed overall visits and telemedicine count among patients with Type 1 diabetes in 2019 and 2020, stratified by age and sex

eTable 5. Observed overall visits and telemedicine count among patients with Type 2 diabetes in 2019 and 2020, stratified by age and sex

eTable 6. Difference-in-difference model: Total and telemedicine visits in 2020 versus 2019 for patients with type 1 diabetes, stratified by each complication as defined by the Diabetes Complications Severity Index score

eTable 7. Difference-in-difference model: Total and telemedicine visits in 2020 vs. 2019 for patients with type 2 diabetes, stratified by each complication as defined by the Diabetes Complications Severity Index score

eTable 8. The negative binomial regression models with cluster-robust standard errors for patients with type 1 diabetes

eTable 9. The negative binomial regression models with cluster-robust standard errors for patients with type 2 diabetes
