# Supplementary material for: Influence of the COVID-19 Pandemic on Overall Physician Visits and Telemedicine Use Among Patients With Type 1 or Type 2 Diabetes in Japan
Source: J Epidemiol. 2022 Oct 5;32(10):476–82. doi: 10.2188/jea.JE20220032 (PMC9424188; doi:10.2188/jea.JE20220032)
Supplement: Supplementary file 1 [file je-32-476-s001.pdf]

eTable 1. Medications prescribed codes (WHO-ATC[a] Classification code) for hypertension and dyslipidaemia

Disease	**WHO-ATC Classification code**
Hypertension	Starting with C02
	Starting with C03
	Starting with C07
	Starting with C08
	Starting with C09
Dyslipidaemia	Starting with C10

[a]WHO-ATC, World Health Organization-Anatomical Therapeutic Chemical.

eTable 2. Medical practice codes for self-monitoring of blood glucose levels in patients with Type 1 diabetes or childhood hypoglycaemia[a]

Procedure name (Japanese)	English procedure name (translated by authors)	Procedure code
血糖自己測定器加算(20 回以上)（1 型糖尿病・小児低血糖症等）	Payment for use of self-monitoring of blood glucose device (≥20) (eg, Type 1 diabetes or childhood hypoglycaemia)	114009910
血糖自己測定器加算(30 回以上)（1 型糖尿病・小児低血糖症等）	Payment for use of self-monitoring of blood glucose device (≥30) (eg, Type 1 diabetes or childhood hypoglycaemia)	114046110
血糖自己測定器加算(40 回以上)（1 型糖尿病・小児低血糖症等）	Payment for use of self-monitoring of blood glucose device (≥40) (eg, Type 1 diabetes or childhood hypoglycaemia)	114010010
血糖自己測定器加算(60 回以上)（1 型糖尿病・小児低血糖症等）	Payment for use of self-monitoring of blood glucose device (≥60) (eg, Type 1 diabetes or childhood hypoglycaemia)	114010110
血糖自己測定器加算(90 回以上)（1 型糖尿病・小児低血糖症等）	Payment for use of self-monitoring of blood glucose device (≥90) (eg, Type 1 diabetes or childhood hypoglycaemia)	114010210
血糖自己測定器加算(120 回以上)（1 型糖尿病・小児低血糖症等）	Payment for use of self-monitoring of blood glucose device (≥120) (eg, Type 1 diabetes or childhood hypoglycaemia)	114015610

[a] Procedure codes used in 2018.

eTable 3. Medical practice codes for medical visits

Procedure name (Japanese)	English procedure name (translated by authors)	Procedure code	Counted as overall outpatient visits	Counted as telemedicine use
初診料	First consultation fee	111000110	Yes	No
初診料（文章による紹介がない患者）	First consultation fee (without written referral)	111012510	Yes	No
初診料（新型コロナウイルス感染症・診療報酬上臨時取扱）	First consultation fee (temporary code for COVID-19)	111013850	Yes	No
再診料	Follow-up consultation fee	112007410	Yes	No
同日再診	Follow-up consultation fee (same day)	112008150	Yes	No
外来診療料	Follow-up consultation fee for large-scale hospitals	112016310	Yes	No
生活習慣病管理料（処方せんを交付糖尿病を主病）	Lifestyle-related disease management fee (prescriptions issued, diabetes as the main disease)	113005910	Yes	No
電話等再診	Consultation by telephone, etc. (telemedicine)	112007950	Yes	Yes
オンライン診療料 [a]	Online consultation fee (telemedicine)	112023210	Yes	Yes

[a] The telemedicine code deregulated during emergency declaration is 112007950.

eTable 4. Observed overall visits and telemedicine count among patients with Type 1 diabetes in 2019 and 2020, stratified by age and sex

		Observed visit counts per 100 patients														
		Overall (n=4,582)					Men (n=2,473)					Women (n=2,109)				
	Year	Jan	Feb	Mar	Apr	May	Jan	Feb	Mar	Apr	May	Jan	Feb	Mar	Apr	May
**Total overall visits**																
Total	2019	77.15	74.81	78.77	79.25	75.25	76.26	75.21	79.22	80.19	74.53	78.19	74.35	78.24	78.14	80.84
	2020	76.87	74.99	78.22	76.50	66.24	75.90	74.89	79.22	76.43	68.78	78.00	75.11	77.05	76.58	63.25
<19 years	2019	83.72	85.47	88.37	84.30	87.40	81.82	85.65	87.08	85.65	86.12	85.02	85.34	89.25	83.39	88.27
	2020	86.43	81.01	87.21	75.39	70.93	82.30	80.86	87.08	75.60	73.68	89.25	81.11	87.30	75.24	69.06
20–39 years	2019	74.05	72.51	75.68	74.59	70.89	70.79	72.34	74.74	74.40	70.10	77.67	72.71	76.72	74.81	71.76
	2020	75.05	71.07	76.04	73.87	62.39	71.65	68.56	75.60	71.65	63.92	78.82	73.86	76.53	76.34	60.69
40–59 years	2019	76.44	73.17	78.30	79.43	73.58	77.20	74.20	79.79	81.40	73.43	75.41	71.77	76.27	76.75	73.78
	2020	76.20	74.71	77.33	76.81	66.75	76.57	76.36	79.09	77.97	69.23	75.69	72.44	74.93	75.22	63.35
≥60 years	2019	80.83	77.11	77.94	83.51	80.83	78.97	78.97	79.76	82.14	81.35	82.83	75.11	75.97	84.98	80.26
	2020	74.23	78.97	78.14	82.06	67.42	76.59	76.19	81.75	79.37	73.41	71.67	81.97	74.25	84.98	60.94
**Telemedicine**																
Total	2019	0.00	0.00	0.00	0.00	0.00	0.00	0.00	0.00	0.00	0.00	0.00	0.00	0.00	0.00	0.00
	2020	0.00	0.00	0.09	0.74	0.57	0.00	0.00	0.04	0.61	0.65	0.00	0.00	0.14	0.90	0.47
<19 years	2019	0.00	0.00	0.00	0.00	0.00	0.00	0.00	0.00	0.00	0.00	0.00	0.00	0.00	0.00	0.00
	2020	0.00	0.00	0.19	0.78	0.58	0.00	0.00	0.00	1.44	0.96	0.00	0.00	0.33	0.33	0.33
20–39 years	2019	0.00	0.00	0.00	0.00	0.00	0.00	0.00	0.00	0.00	0.00	0.00	0.00	0.00	0.00	0.00
	2020	0.00	0.00	0.09	0.72	0.72	0.00	0.00	0.17	0.52	1.03	0.00	0.00	0.00	0.95	0.38
40–59 years	2019	0.00	0.00	0.00	0.00	0.00	0.00	0.00	0.00	0.00	0.00	0.00	0.00	0.00	0.00	0.00
	2020	0.00	0.00	0.08	0.81	0.57	0.00	0.00	0.00	0.56	0.49	0.00	0.00	0.19	1.15	0.67
≥60 years	2019	0.00	0.00	0.00	0.00	0.00	0.00	0.00	0.00	0.00	0.00	0.00	0.00	0.00	0.00	0.00
	2020	0.00	0.00	0.00	0.41	0.21	0.00	0.00	0.00	0.40	0.40	0.00	0.00	0.00	0.43	0.00

eTable 5. Observed overall visits and telemedicine count among patients with Type 2 diabetes in 2019 and 2020, stratified by age and sex

		Observed visits per 100 patients														
		Overall (n=120,631)					Men (n=90,515)					Women (n=30,116)				
	Year	Jan	Feb	Mar	Apr	May	Jan	Feb	Mar	Apr	May	Jan	Feb	Mar	Apr	May
**Total overall visits**																
Total	2019	75.74	73.70	79.51	80.76	73.33	71.90	70.29	76.29	76.72	69.20	75.56	72.45	77.26	80.06	74.02
	2020	73.14	74.72	74.95	76.21	67.54	69.45	71.80	71.49	72.71	65.01	72.85	71.93	73.66	74.78	64.94
20–39 years	2019	70.22	69.39	71.59	73.44	64.19	69.85	68.60	72.00	73.00	63.70	71.30	71.75	70.42	74.72	65.61
	2020	63.34	65.75	65.91	65.83	59.82	63.26	66.54	66.35	66.02	60.74	63.59	63.46	64.67	65.23	57.14
40–59 years	2019	75.53	73.17	79.91	80.35	72.67	74.10	72.15	79.27	78.93	70.96	80.35	76.63	82.05	85.12	78.44
	2020	72.79	74.77	74.53	76.13	67.72	71.40	74.32	73.57	75.10	67.45	77.46	76.29	77.75	79.53	68.62
60–69 years	2019	76.93	75.39	79.99	82.48	76.04	75.26	74.33	78.54	81.01	74.16	81.18	78.10	83.69	86.22	80.85
	2020	75.50	75.94	77.30	77.94	68.49	74.04	74.94	75.76	76.62	67.32	79.22	78.50	81.23	81.28	71.42
≥70 years	2019	78.61	77.09	80.06	86.14	78.52	76.11	74.27	76.21	82.69	76.18	82.68	81.69	86.31	91.75	82.33
	2020	76.36	77.63	78.41	80.06	68.32	73.73	74.78	75.86	77.33	66.90	80.63	82.27	82.56	84.49	70.63
**Telemedicine**																
Total	2019	0.01	0.00	0.00	0.01	0.00	0.01	0.00	0.00	0.01	0.00	0.01	0.01	0.00	0.01	0.00
	2020	0.00	0.01	0.14	1.19	0.78	0.00	0.01	0.12	1.14	0.75	0.00	0.01	0.20	1.34	0.87
20–39 years	2019	0.02	0.05	0.02	0.05	0.02	0.02	0.02	0.02	0.07	0.02	0.00	0.13	0.00	0.00	0.00
	2020	0.02	0.00	0.11	0.86	0.72	0.02	0.00	0.11	0.66	0.77	0.00	0.00	0.13	1.39	0.57
40–59 years	2019	0.01	0.00	0.00	0.01	0.00	0.01	0.00	0.00	0.00	0.00	0.02	0.01	0.01	0.02	0.01
	2020	0.00	0.01	0.13	1.11	0.73	0.00	0.01	0.10	1.05	0.70	0.01	0.01	0.20	1.31	0.85
60–69 years	2019	0.00	0.00	0.00	0.00	0.00	0.00	0.00	0.00	0.00	0.00	0.00	0.00	0.00	0.00	0.00
	2020	0.00	0.01	0.16	1.38	0.90	0.00	0.01	0.14	1.37	0.87	0.00	0.00	0.21	1.40	0.99
≥70 years	2019	0.00	0.00	0.02	0.00	0.00	0.00	0.00	0.04	0.00	0.00	0.00	0.00	0.00	0.00	0.00
	2020	0.00	0.00	0.27	1.74	0.76	0.00	0.00	0.29	1.98	0.83	0.00	0.00	0.23	1.35	0.64

eTable 6. Difference-in-difference model: Total and telemedicine visits in 2020 versus 2019 for patients with type 1 diabetes, stratified by each complication as defined by the Diabetes Complications Severity Index score

	Number of visits per 100 people with diabetes (95% confidence interval)		
	Overall (n=4.582)		
	Preintervention periods	Apr	May
**Total overall physician visits**			
Retinopathy (+)	0.03 (-2.01 to 2.08)	-4.29 (-8.02 to -0.56)	-11.86 (-15.36 to -8.37)
Retinopathy (-)	-0.43 (-1.89 to 1.03)	-2.04 (-4.62 to 0.54)	-7.17 (-9.73 to -4.60)
Nephrosis (+)	0.48 (-2.20 to 3.15)	0.55 (-4.27 to 5.38)	-11.45 (-16.50 to -6.41)
Nephrosis (-)	-0.26 (-1.55 to 1.03)	-3.43 (-5.77 to -1.10)	-8.74 (-11.00 to -6.49)
Neuropathy (+)	0.24 (-3.13 to 3.61)	-0.58 (-6.75 to 5.59)	-10.76 (-16.58 to -4.93)
Neuropathy (-)	-0.19 (-1.44 to 1.06)	-2.84 (-5.07 to -0.62)	-8.48 (-10.67 to -6.29)
Cerebrovascular disease (+)	6.25 (-21.44 to 33.94)	-25.00 (-91.64 to 41.64)	-25.00 (-86.08 to 36.08)
Cerebrovascular disease (-)	-0.22 (-1.42 to 0.99)	-2.74 (-4.89 to -0.58)	-8.74 (-10.83 to -6.64)
Cardiovascular disease (+)	3.50 (-3.43 to 10.42)	-5.13 (-17.14 to 6.89)	3.96 (-9.36 to 17.28)
Cardiovascular disease (-)	-0.41 (-1.64 to 0.81)	-2.45 (-4.65 to -0.24)	-9.15 (-11.29 to -7.01)
Peripheral vascular disease (+)	-4.55 (-15.09 to 6.00)	6.06 (-12.97 to 25.09)	7.58 (-8.48 to 23.63)
Peripheral vascular disease (-)	-0.22 (-1.40 to 0.97)	-2.95 (-5.08 to -0.82)	-9.29 (-11.37 to -7.21)
**Telemedicine**			
Retinopathy (+)	0.03 (-0.03 to 0.01)	0.58 (0.18–0.97)	0.97 (0.47–1.48)
Retinopathy (-)	0.05 (-0.01 to 0.11)	0.79 (0.46–1.11)	0.34 (0.12–0.57)
Nephrosis (+)	0.00 (0.00–0.00)	1.07 (0.37–1.77)	0.48 (0.01–0.94)
Nephrosis (-)	0.05 (0.00–0.11)	0.65 (0.38–0.91)	0.54 (0.29–0.79)
Neuropathy (+)	0.00 (0.00–0.00)	0.79 (0.10–1.49)	1.11 (0.29–1.94)
Neuropathy (-)	0.05 (0.00–0.10	0.70 (0.43–0.97)	0.45 (0.23–0.67)
Cerebrovascular disease (+)	0.00 (0.00–0.00)	0.00 (0.00–0.00)	0.00 (0.00–0.00)
Cerebrovascular disease (-)	0.05 (0.00–0.09)	0.75 (0.49–1.01)	0.57 (0.34–0.80
Cardiovascular disease (+)	0.00 (0.00–0.00)	0.00 (0.00–0.00)	0.21 (-0.28 to 4.48)
Cardiovascular disease (-)	0.05 (0.00–0.10)	0.75 (0.48–1.01)	0.50 (0.28–0.73)
Peripheral vascular disease (+)	0.00 (0.00–0.00)	0.00 (0.00–0.00)	1.52 (-1.52 to 4.55)
Peripheral vascular disease (-)	0.05 (0.00–0.09)	0.72 (0.46–0.97)	0.54 (0.31–0.76)

eTable 7. Difference-in-difference model: Total and telemedicine visits in 2020 vs. 2019 for patients with type 2 diabetes, stratified by each complication as defined by the Diabetes Complications Severity Index score

	Number of visits per 100 people with diabetes (95% confidence interval)		
		Overall (n=120,631)	
	Preintervention periods	Apr	May
**Total overall physician visits**			
Retinopathy (+)	-1.46 (-2.09 to -0.84)	-3.11 (-4.24 to -1.99)	-5.22 (-6.27 to -4.18)
Retinopathy (-)	-0.87 (-1.15 to -0.59)	-2.37 (-2.87 to -1.86)	-3.40 (-3.86 to -2.94)
Nephrosis (+)	-1.26 (-1.91 to -0.62)	-2.54 (-3.69 to -1.39)	-3.63 (-4.71 to -2.55)
Nephrosis (-)	-0.99 (-1.27 to -0.72)	-2.48 (-2.98 to -1.99)	-3.59 (-4.04 to -3.13)
Neuropathy (+)	-1.26 (-2.29 to -0.23)	-1.52 (-3.43 to 0.39)	-6.09 (-7.86 to -4.32)
Neuropathy (-)	-0.97 (-1.23 to -0.70)	-2.57 (-3.03 to -2.10)	-3.57 (-4.00 to -3.14)
Cerebrovascular disease (+)	4.46 (0.11–8.81)	3.30 (-6.01 to 12.61)	-4.62 (-12.96 to 3.72)
Cerebrovascular disease (-)	-1.04 (-1.30 to -0.78)	-2.35 (-2.83 to -1.88)	-3.46 (-3.90 to -3.02)
Cardiovascular disease (+)	-1.16 (-2.26 to -0.06)	-3.28 (-5.24 to -1.33)	-5.38 (-7.17 to -3.60)
Cardiovascular disease (-)	-1.09 (-1.36 to -0.81)	-2.21 (-2.71 to -1.71)	-3.34 (-3.81 to -2.88)
Peripheral vascular disease (+)	-0.08 (-2.82 to 2.66)	-2.00 (-6.94 to 2.94)	-5.78 (-10.34 to -1.22)
Peripheral vascular disease (-)	-1.01 (-1.26 to -0.75)	-2.47 (-2.93 to -2.02)	-3.69 (-4.12 to -3.27)
**Telemedicine**			
Retinopathy (+)	0.06 (0.04–0.09)	1.16 (1.01–1.31)	0.70 (0.58–0.82)
Retinopathy (-)	0.07 (0.06–0.08)	1.14 (1.07–1.20)	0.74 (0.68–0.80)
Nephrosis (+)	0.07 (0.04–0.10)	1.17 (1.01–1.33)	0.65 (0.53–0.77)
Nephrosis (-)	0.07 (0.06–0.08)	1.14 (1.07–1.20)	0.75 (0.69–0.80)
Neuropathy (+)	0.08 (0.03–0.13)	1.17 (0.93–1.41)	0.56 (0.39–0.73)
Neuropathy (-)	0.07 (0.06–0.08)	1.13 (1.07–1.20)	0.74 (0.69–0.79)
Cerebrovascular disease (+)	0.17 (-0.16 to 0.49)	0.22 (-0.47 to 0.91)	1.21 (-0.10 to 2.52)
Cerebrovascular disease (-)	0.07 (0.06–0.08)	1.12 (1.06–1.19)	0.72 (0.67–0.77)
Cardiovascular disease (+)	0.06 (0.02–0.10)	0.96 (0.72–1.20)	0.68 (0.48–0.89)
Cardiovascular disease (-)	0.07 (0.06–0.08)	1.16 (1.09–1.23)	0.75 (0.70–0.81)
Peripheral vascular disease (+)	0.12 (-0.02 to 0.26)	0.90 (0.31–1.50)	0.90 (0.34–1.47)
Peripheral vascular disease (-)	0.07 (0.06–0.08)	1.14 (1.08–1.21)	0.73 (0.68–0.78)

eTable 8. The negative binomial regression models with cluster-robust standard errors for patients with type 1 diabetes

	Number of visits per 100 people with diabetes (95% confidence interval) [a]					
	Overall (n=4,582)		Men (n=2,473)		Women (n=2,109)	
	Apr [a]	May [a]	Apr [a]	May [a]	Apr [a]	May [a]
**Total overall physician visits**						
Total	-2.53 (-4.63 to -0.44)	-8.80 (-10.85 to -6.74)	-3.53 (-6.43 to -0.62)	-5.51 (-8.34 to -2.68)	-1.36 (-4.38 to 1.66)	-12.64 (-15.6 to -9.68)
1–19 years	-7.95 (-13.43 to -2.46)	-15.50 (-21.63 to -9.38)	-8.61 (-16.94 to -0.29)	-11.00 (-20.52 to -1.49)	-7.49 (-14.78 to -0.20)	-18.57 (-26.56 to -10.57)
20–39 years	-0.69 (-4.80 to 3.42)	-8.47 (-12.64 to -4.30)	-2.06 (-7.82 to 3.70)	-5.50 (-11.33 to 0.34)	0.82 (-5.05 to 6.70)	-11.76 (-17.72 to -5.81)
40–59 years	-2.73 (-5.67 to 0.21)	-6.93 (-9.72 to -4.15)	-3.70 (-7.65 to 2.33)	-4.48 (-8.18 to -0.77)	-1.40 (-5.81 to 3.00)	-10.30 (-14.49 to -6.10)
≥60 years	0.07 (-6.61 to 6.75)	-11.89 (-18.24 to -5.53)	-17.20 (-11.20 to 7.76)	-68.78 (-160.97 to 23.40)	2.00 (-7.40 to 11.41)	-17.31 (-25.96 to -8.65)

[a] The estimates for April or May 2020 represent ([number of visits in April or May 2020] - [average number of visits per month between January and March 2020]) －([number of visits in April or May 2019] - [average number of visits per month between January and March 2019]).

eTable 9. The negative binomial regression models with cluster-robust standard errors for patients with type 2 diabetes

	Number of visits per 100 people with diabetes (95% confidence interval) [a]					
	Overall (n=120,631)		Men (n=90,515)		Women (n=30,116)	
	Apr [a]	May [a]	Apr [a]	May [a]	Apr [a]	May [a]
**Total overall physician visits**						
Total	-2.50 (-2.95 to -2.04)	-3.74 (-4.16 to -3.32)	-2.23 (-2.75 to -1.70)	-2.54 (-3.02 to -2.04)	-3.31 (-4.22 to -2.41)	-7.36 (-8.20 to -6.53)
20–39 years	-2.21 (-4.24 to -0.18)	1.02 (-0.87 to 2.91)	-2.20 (-4.53 to 0.13)	1.80 (-0.38 to 3.98)	-2.23 (-6.34 to 1.87)	-1.22 (-4.99 to 2.54)
40–59 years	-2.04 (-2.60 to -1.48)	-2.77 (-3.30 to -2.25)	-1.73 (-2.38 to -1.09)	-1.42 (-2.02 to -0.83)	-3.06 (-4.23 to -1.89)	-7.31 (-8.40 to -6.22)
60–69 years	-3.35 (-4.23 to -2.46)	-6.37 (-7.17 to -5.57)	-3.25 (-4.30 to -2.20)	-5.70 (-6.64 to -4.75)	-3.60 (-5.23 to -1.96)	-8.07 (-9.57 to -6.57)
≥70 years	-4.96 (-7.28 to -2.64)	-9.08 (-11.22 to -6.95)	-4.61 (-7.61 to -1.62)	-8.54 (-11.28 to -5.80)	-5.52 (-9.18 to -1.86)	-10.00 (-13.37 to -6.56)

[a] The estimates for April or May 2020 represent ([number of visits in April or May 2020] - [average number of visits per month between January and March 2020]) －([number of visits in April or May 2019] - [average number of visits per month between January and March 2019]).
